# Optimal Design and Biomechanical Analysis of a Biomimetic Lightweight Design Plate for Distal Tibial Fractures: A Finite Element Analysis

**DOI:** 10.3389/fbioe.2022.820921

**Published:** 2022-02-21

**Authors:** Mian Wang, Yuping Deng, Pusheng Xie, Jinchuan Tan, Yang Yang, Hanbin Ouyang, Dongliang Zhao, Gang Huang, Wenhua Huang

**Affiliations:** ^1^ National Key Discipline of Human Anatomy, Guangdong Provincial Key Laboratory of Medical Biomechanics, Guangdong Engineering Research Center for Translation of Medical 3D Printing Application, School of Basic Medical Sciences, Southern Medical University, Guangzhou, China; ^2^ Institute of Biomedical Engineering, Shenzhen Bay Laboratory, Shenzhen, China; ^3^ Department of Orthopedics and Traumatology, Integrated Hospital of Traditional Chinese Medicine, Southern Medical University, Guangzhou, China; ^4^ Guangdong Medical Innovation Platform for Translation of 3D Printing Application, The Third Affiliated Hospital of Southern Medical University, Guangzhou, China; ^5^ Orthopaedic Center, Affiliated Hospital of Guangdong Medical University, Guangdong Medical University, Zhanjiang, China; ^6^ Drug Discovery Center, State Key Laboratory of Chemical Oncogenomics, School of Chemical Biology and Biotechnology, Peking University Shenzhen Graduate School, Shenzhen, China

**Keywords:** distal tibial fractures, internal fixation, low-profile, plate, topology optimization

## Abstract

The treatment of fractures of the distal tibia can be problematic due to the insubstantial soft-tissue covering this part of the anatomy. This study investigates a novel strategy for minimally invasive plate osteosynthesis of distal tibia fractures called bionic lightweight design plating. Following the structure of the animal trabecular bone, we utilized topological mathematical methods to redesign the material layout of the internal fixation device to fulfill the desired lightweight design within given boundary conditions. The results showed that this method can maintain the same stability of the construct as the original plate after a reduction in the original volume by 30%, and the differences in strain energy of plates and maximum node displacement of constructs between the constructs [RP construct *vs.* LP construct] were not statistically significant (*p* > 0.05). In the safety assessment of the constructs, the peak stress of plates between constructs was found to not be statistically significantly different under a doubled physiological load (*p* > 0.05). The average stress of the plates’ elements exceeding the allowable stress was analyzed, and no statistically significant differences were found between the two constructs under axial compression stress conditions (*p* > 0.05). The average stress of the plates’ elements in the redesigned plating construct under torsional stress conditions was 3.08% less than that of the locked plating construct (*p* < 0.05). Under the double physiological load condition, 89% of the elements of the plate in the redesigned plating construct and 85% of the elements of the plate in the locked plating construct were lower than the maximum safe stress of the plate, which was 410 MPa (secondary allowable stresses). That reminds us the topology optimization offer a possible way to improve the capacity of soft tissue protection while ensuring the safety of the RP construct by reducing the volume of the implants.

## Introduction

The incidence of distal tibia fracture is high, and it is one of the most common long bone fractures (Court-Brown and McBirnie, 1995; Weiss et al., 2009; Vaienti et al., 2019). Such fractures usually lead to the stripping of the periostea, poor soft-tissue blood supply, and severe trauma to the surrounding soft tissue structures because of its relative thinness. Open reduction and internal fixation (ORIF) surgery is suitable for most types of fractures and fracture types that require fine reduction, at the cost of more extensive soft tissue dissection and blood supply effects. In cases of unhealthy soft tissue at the anterior tibia, ORIF surgery on the tibia distal segment can cause increased rates of nonunion, deep infections (Sirkin et al., 2004), and surgical wound dehiscence (Teeny and Wiss, 1993; Sheerin et al., 2006; Vidović et al., 2015). This is true even in a staged approach, which is considered to be an effective way of handling soft tissues (Sheerin et al., 2006; Chan et al., 2017) for distal tibia fractures through temporized treatment. Such techniques protect the affected tissue from iatrogenic trauma through delayed or elective surgery; however, they can also lead to poor outcomes due to the necrosis of the edges of the operative wounds and higher rates of infection following surgical delays greater than 7 days (Mont et al., 1992). However, the timing of surgery in the setting of fractures still largely relies on the experience of the orthopedist (Chou and Lee, 2009).

Work has been underway to develop plating technologies for soft tissue protection (Krackhardt et al., 2005; Pallister and Iorwerth, 2005; Namazi and Mozaffarian, 2007; Cheng et al., 2011; Zhou et al., 2015). However, these efforts have not yielded clinically viable solutions. For example, a minimally invasive plate osteosynthesis (MIPO) technique, based on the less invasive stabilization system and the locking compression plate system, has been developed that enables indirect reduction and stable fixation with minimal surgical trauma relative to ORIF (Vidović et al., 2015). MIPO provides indirect reduction of and minimizes trauma to the soft tissue and maintains the periosteal blood supply, so it should provide undisturbed union and a low rate of complications. However, in distal tibial fractures, reports of delayed union, malunion, skin impingement, and saphenous nerve and vein injury continue to appear with the use of MIPO (Khoury et al., 2002; Lau et al., 2008; Cheng et al., 2011). Further, to minimize the surgical trauma, it is nearly impossible to emphasize an anatomic reduction of the articular surface of a distal fracture of the tibia with intra-articular fractures, which are necessary for the treatment of these distal tibial fractures.

The present study established a theoretical model for evaluating the impact of internal fixation on surrounding soft tissues and investigated a novel strategy for the MIPO of distal tibia fractures called here bionic lightweight design plating. Bionic lightweight design plates utilize topological mathematical models of the number and density of the animal trabecular bone structure changing with the stress distributions which redesign the material layout of the address area of the internal fixation device to develop a lightweight design that would be desirable under a series of boundary conditions. Using finite element (FE) methods, the designer can access a wealth of valuable information for use in medical implant redesign with minimal expense of material resources and funds. Although the topological optimization associated with the FE method can generate a new, customized, and optimized plate design for tibia distal fractures, this has not yet been utilized or reported on. We hypothesized that bionic lightweight design optimization plates will reduce the compression of the implant on the soft tissue by reducing the implant volume while retaining construct strength relative to standard locking plates.

## Materials and Methods

### Geometrical Modelling

A tibia structure model was reconstructed using Mimics 14.0 software (Materialise, Leuven, Belgium) from a series of computed tomography images (Optima CT660, GE medical systems, USA) of a healthy 27-year-old male volunteer (with fully understand the purpose of the data and sign the informed consent document). The whole tibia, distal femoral condyle, and proximal talar condyle were scanned. The slice thickness was 1.25 mm and the plane resolution was 512 × 512 pixels. All procedures were approved by the Ethical Inspection Committee of Integrated Hospital of Traditional Chinese Medicine. This model was imported into the Unigraphics NX 8.5 software (Siemens, Munich, Germany) to build an AO-43C3 type distal tibial fracture according to the clinical fracture classification of association for the study of internal fixation. The tibia was divided into two major fragments with a fracture gap of 3 mm. The gap was located 40 mm above the tibiotalar joint.

The computer aided design model of a general six-hole locking plate (LP) is 3.2 mm thick (LCP Distal Tibia Plates, 6-Hole, Synthes) and uses 3.5 mm diameter screws to fix the LP to the tibia. As this LCP distal tibia plates are most commonly used in clinic to treat AO-43C3 type tibial fracture. We adopted this LP construct as a prototype and optimized on this basis.

**FIGURE 1:**
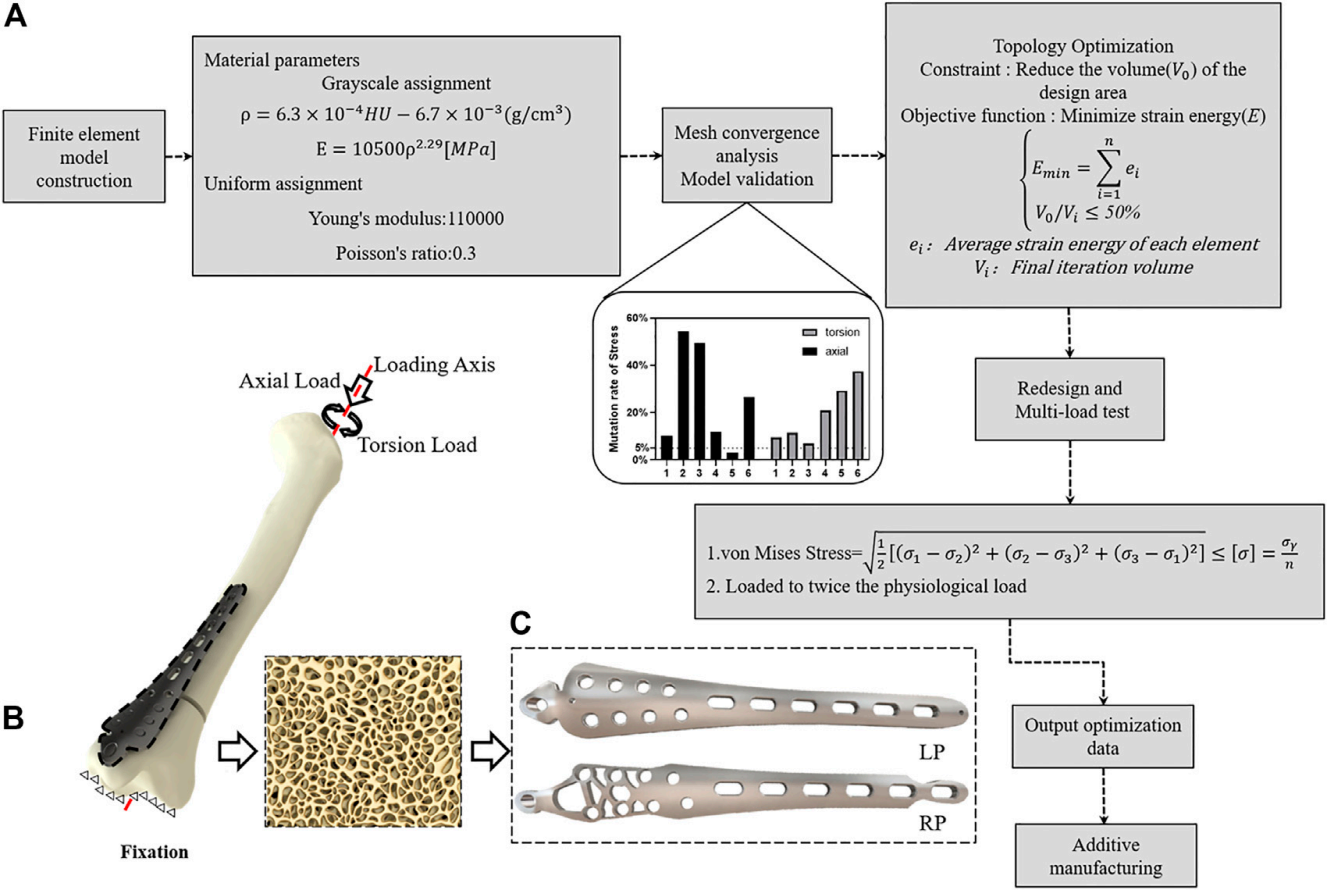
Construction of lightweight design plates inspired by trabecular bone biomechanical responses. **(A)** Topological optimization technical route. **(B)** Finite element simulation boundary conditions. **(C)** Implantation shape before and after optimization.

Following previous biomechanical studies, we mimic the experimental setup with the proximal tibia embedded centrally in an embedding box with 10-mm diameter loading disks to validate the simulation model (Hoegel et al., 2012; Högel et al., 2012). The positional relationship between the implant and the tibia and the boundary conditions of whole model are shown in (Figure 1B).

The two models were imported into Abaqus 2017 (Dassault Systems, Velizy-Villacoublay, France) for FE modelling. The mesh densities were settled to 1.1 mm for the LP model followed convergence analyses within the capacity of the available computers. Then 10-node tetrahedral elements were used to discretize all of the parts of the bone-implant system. The details of these elements are given in Table 1.

**TABLE 1:** Types of elements, number of nodes, and number of elements in each part of the model.

Parts	Element type	Number of elements	Number of nodes
Tibia	C3D10	56,804	84,837
Screw	C3D10	269,041	175,364
Locking Plate	C3D10	142,407	215,871
Redesigned Plate	C3D10	419,629	624,277

### Mechanical Environment Theoretical Model Analyses of Post-operation Soft Tissue

**FIGURE 2:**
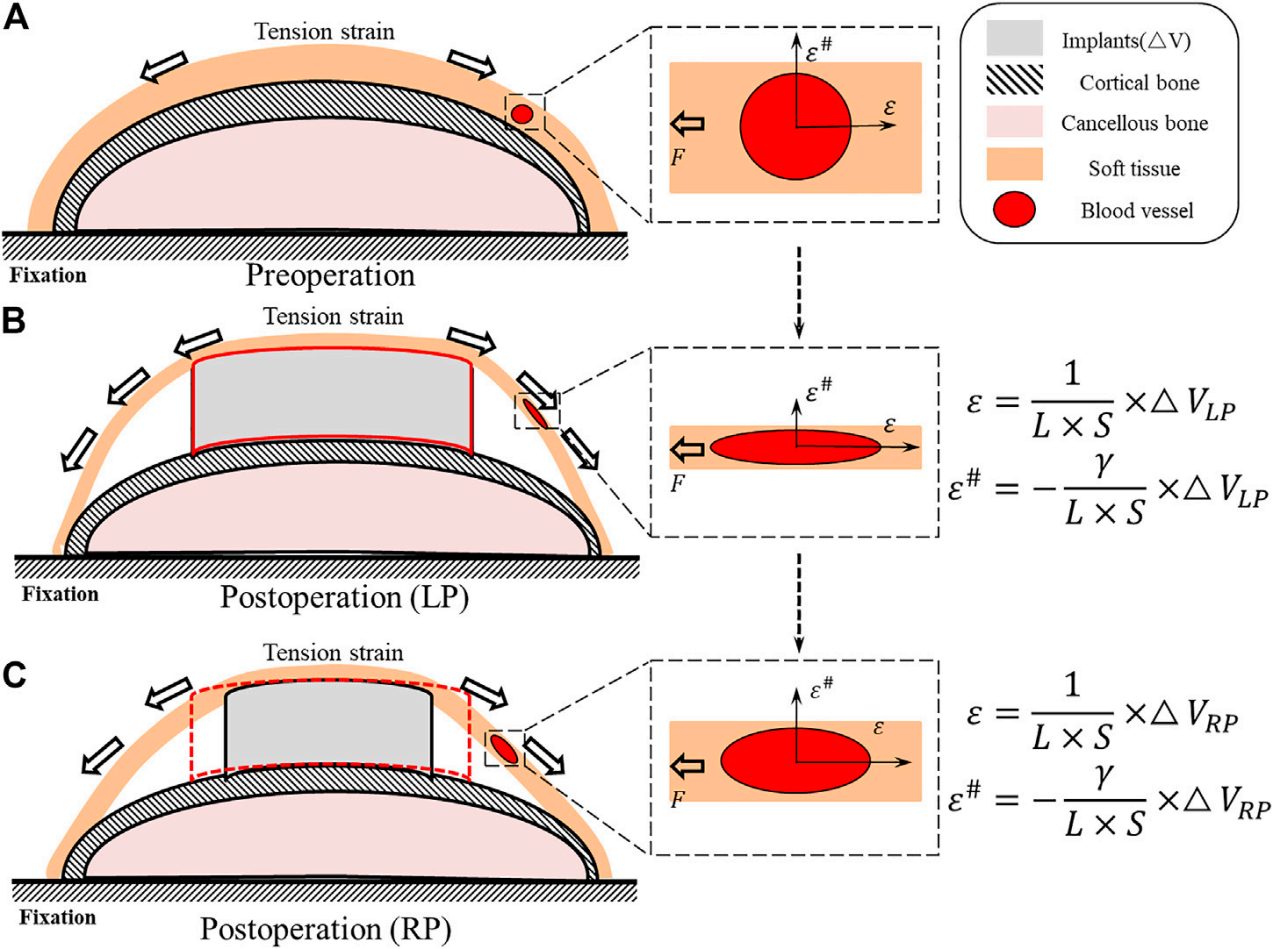
Schematic diagram of the internal implantation of the distal tibia fracture implant. **(A)** The relative position and morphology of the tibia and the soft tissue of the skin and the blood vessels passing through it under physiological conditions. **(B)** The strain increases after LP implantation and causes the strain of the strain direction ε to increase and the strain in the vertical direction $\varepsilon^{\#}\;$ to increase. This leads the soft tissue and blood vessels to stretch with the affected blood the supply. **(C)** After implantation, with smaller  ΔV , the strain of the strain direction ε is reduced, and the strain in the vertical direction $\;\varepsilon^{\#}\;$ is reduced, and the deformation of the soft tissue and blood vessels is reduced.

The structural distribution of each part after the placement of the implants is presented in Figure 2. The original position of the entire soft tissue is occupied by the implant, and the relative position of the soft tissue changes (Figures 2B,C). Assuming that the soft tissue is a beam model, the implantation of the internal fixation causes the beam to be stretched; specifically, the equivalent volume of the internal fixation implant V can be calculated with the following equation:

V=L×S (1)

where S represents the cross-sectional area of internal fixation, and L represents the unit thickness of the internal fixation; the mechanical effect on the soft tissue after implantation is approximated as a stretch model, and the strain of the strain direction ε  can be expressed as follows:

$$\varepsilon =\frac{\Delta V}{L\times S}$$ (2)

where ΔV represents the volume change with or without internal fixation. The following equation represents the tissue tensile balance force:

F=σ×A (3)

where *F* is the tensile force on the section, σ represents the stress on the section, and A represents the cross-sectional area of the soft tissue, which can also be written as:

F=E×ε×A (4)

Combining Eq. 4 with Eq. 2 gives:

$$F=E\times A\times \frac{\Delta V}{L\times S}$$ (5)

where in Eqs 4, 5 the *E* represents the soft tissue elastic modulus. On the other hand, Poisson’s ratio *γ* in the theoretical model can be written as:

$$\gamma =-\frac{\varepsilon^{\#}}{\varepsilon }$$ (6)

And therefore can be written as:

$$\varepsilon^{\#}=-\gamma \times \varepsilon$$ (7)

Combining Eq. 7 with Eq. 2 gives:

$$\varepsilon^{\#}=-\frac{\gamma }{L\times S}\times \Delta V$$ (8)

Where $\varepsilon^{\#}$ represents the strain in the vertical direction; Eqs 2, 3 can be combined with (Eq. 4) to be written as a formula for σ :

$$\sigma =\frac{E}{L\times S}\times \Delta V$$ (9)

### Material Properties

The tibia was modeled as an elastic and non-homogenous material by assigning specific Young’s modulus values to each element using Mimics. To calculate each element’s modulus of elasticity, the Hounsfield unit was extracted from CT images and calculated at the centroid of each element. Formulas 10 and 11 were used in previous studies (Chen et al., 2010; Nourisa and Rouhi, 2016; Xie et al., 2020) to find the apparent density (ρ) and Young’s modulus (*E*) of each element.

$$\rho =6.3\times 10^{-4}HU-6.7\times 10^{-3}\left[\frac{g}{cm^{3}}\right]$$ (10)

$$E=10500\rho^{2.29}\left[MPa\right]$$ (11)

The materials of the LP and the screws were assumed to be titanium alloy (Ti6Al4V). Both the embedding box and loading disk were considered rigid during the analysis.

### Boundary and Loading Conditions

Previous work (Cristofolini and Viceconti, 2000; Stoffel et al., 2004; Hoegel et al., 2012) has fully constrained the distal tibialis-articular surface, and the proximal tibia was bonded with the embedding box. The engagement of the screw-plate interface was completely bonded to all screws and to the screw-bone interface. The direction of the loading was along the tibial mechanical axis.

For optimization, the construct was simulated in the two-leg stance, in which the patient tries to load both sides equally. We considered a load equal to 50% of the body weight of a 70 kg person (F = 350 N), and we set a torque of 3500 Nmm along the internal direction of rotation of the tibial mechanical axis as the physiological loading condition (Hoegel et al., 2012; Högel et al., 2012).

To test the stability and safety of the bionic lightweight design constructs, a series of loads were placed in succession on the loading disk. For axial compression stress, loads ranged from 50 to 700 N with increments of 50 N, and for torsion stress, the loads ranged from 1000 to 7000 N·mm at increments of 500 N·mm (Snow et al., 2008) (Table 2).

**TABLE 2:** Loading conditions and load values corresponding to each construct of LP and RP e.g., LP-A350 means the LP construct uses 350 N as the load value under axial compression loading conditions.

Axial compression			Torsion		
Load (N)	LP	RP	Load (N·mm)	LP	RP
50	LP-A50	RP-A50	1000	LP-T100	RP-T100
100	LP-A100	RP-A100	1500	LP-T150	RP-T150
150	LP-A150	RP-A150	2000	LP-T200	RP-T200
200	LP-A200	RP-A200	2500	LP-T250	RP-T250
250	LP-A250	RP-A250	3000	LP-T300	RP-T300
300	LP-A300	RP-A300	3500	LP-T350	RP-T350
350	LP-A350	RP-A350	4000	LP-T400	RP-T400
400	LP-A400	RP-A400	4500	LP-T450	RP-T450
450	LP-A450	RP-A450	5000	LP-T500	RP-T500
500	LP-A500	RP-A500	5500	LP-T550	RP-T550
550	LP-A550	RP-A550	6000	LP-T600	RP-T600
600	LP-A600	RP-A600	6500	LP-T650	RP-T650
650	LP-A650	RP-A650	7000	LP-T700	RP-T700
700	LP-A700	RP-A700	—	—	—

### Optimization of the Tibia Locking Plate

The optimization of the distal tibia plate occurred in two steps: topology optimization and plate redesign. Initially, the LP model served as a template for topology optimization. Then, design variable, objective function, and constraints were predefined as essential parameters. In this study, the strategy of topology optimization was to minimize the strain energy of the LP model to achieve maximal fixation stiffness as the objective function. The optimal solution is determined by iteratively changing the element density within predetermined constraints (Ouyang et al., 2017). The 50% volume of LP was fixed as the optimization constraint of the redesigned plate (RP). The optimization strategy minimizes the structural compliance of the plate while satisfying the volume of the structure and can be stated as:

$$\left\{ \begin{array}{l} E_{min}=\sum_{i=1}^{n}e_{i}\\ V_{0}/V_{i}\leq 50\% \end{array}\right.$$ (12)

$e_{i}$ : Average strain energy of each element.

$V_{i}$ : Final iteration volume.

Several iterations of the optimization of the material layout were performed with the goal of maximizing the performance of the system for use as the basis for the design of the RP. The RP was designed to have the same thickness and load and to be in the same configuration as in the LP model. Subsequently, the RP model was analyzed and compared to the LP construct (Figure 1C).

### Statistical Methods

To qualify the stability and safety of the topological optimization and the plate redesign, we compared the differences in biomechanical behavior between 15 loads on the plates in the LP constructs and the RP constructs, particularly in terms of fixed stability, including 1) the strain energy of the fixation models 2) and the construct stiffness of the plate fixation system for each load. The safety assessment included 1) the von Mises stress distribution and peak and average values of the plates in both the LP and the RP constructs and 2) the von Mises stress distribution ratio of all elements of the plates in the RP and the LP constructs.

All of the data were collected from the plate elements of the RP and LP constructs. The software SPSS Statistics v. 20 (IBM, Armonk, NY, USA) was used to statistical analysis. The box plots (Figures 4B,D) represent the mean, and the error bars indicate maximum and minimum values. Descriptive statistics and independent-sample t tests were used in the analyses to compare the means. For all statistical analyses,  *P< 0.05  was considered to indicate significance.

## Results

### Theoretical Model Analysis

The positive correlation between *F* and ΔV can be obtained using Eq. 5. Additionally, without considering the change in *A*, the deformation of extension is also positively correlated with ΔV . Further, it can be concluded from Eq. 8 that $\;\varepsilon^{\#}$ increases with the increase of ΔV .

The RP construct and the LP construct volume as $\Delta V_{RP}=3159.61{\text{ mm}}^{3}$ and $\Delta V_{LP}=4481.82{\text{ mm}}^{3}$ are placed into formula (2). Eqs 8, 9 can represent the relationship between ΔV and the strain in the vertical direction $\;\varepsilon^{\#}$ ; the strain in the direction of strain ε and the stress on the section  σ are described in Table 3.

**TABLE 3:** Relationship between stress strain and volume in theoretical model.

*σ*	ε	$\varepsilon^{\#}$
$\Delta V_{LP}$ $\sigma =\frac{E}{L\times S}\times 4481.82$	$\varepsilon =\frac{1}{L\times S}\times 4481.82$	$\varepsilon^{\#}=-\frac{\gamma }{L\times S}\times 4481.82$
$\Delta V_{RP}$ $\sigma =\frac{E}{L\times S}\times 3159.61$	$\varepsilon =\frac{1}{L\times S}\times 3159.61$	$\varepsilon^{\#}=-\frac{\gamma }{L\times S}\times 3159.61$

### Fixation Stability

**FIGURE 3:**
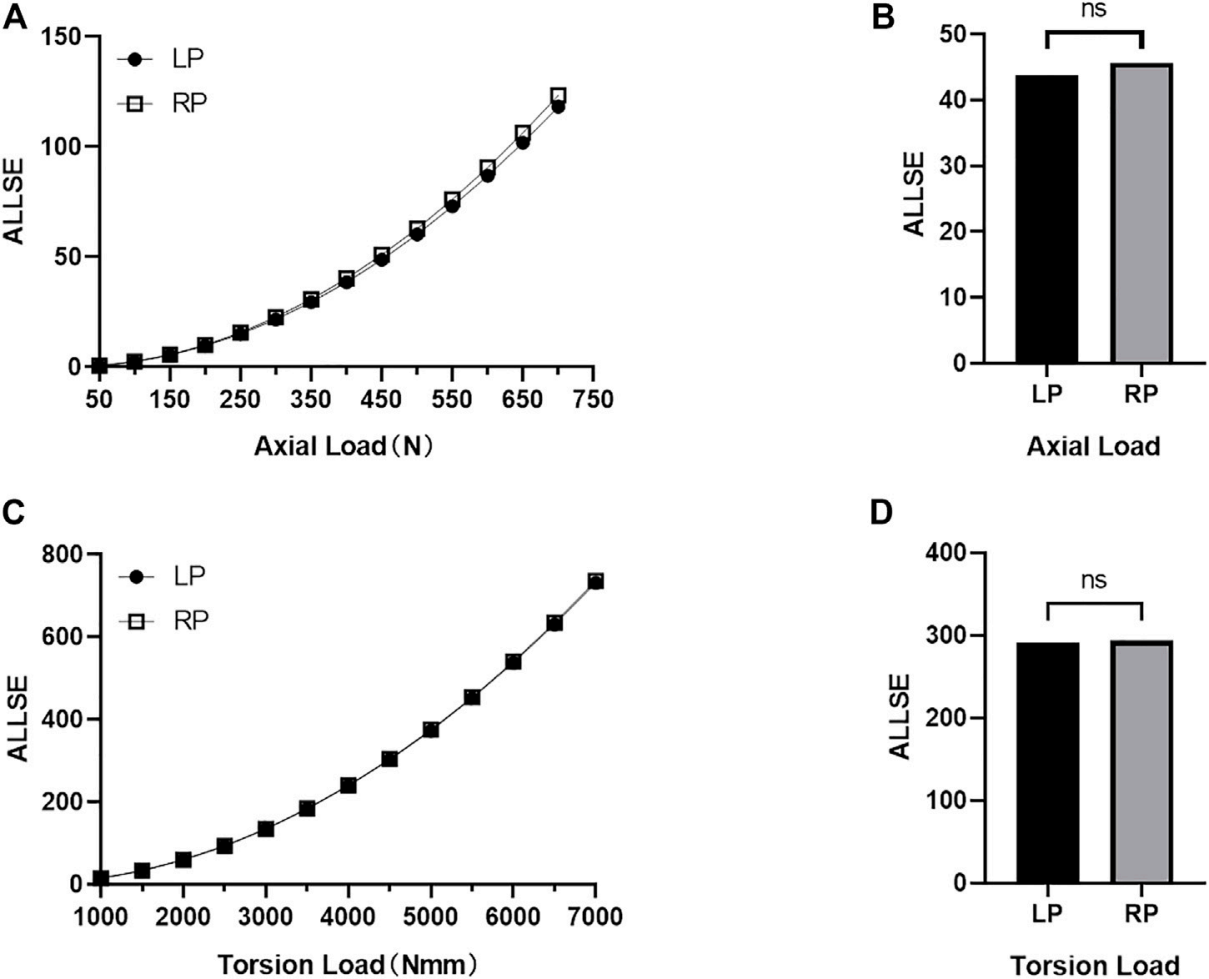
The strain energy (ALLSE) of whole the LP and RP construct changes with the load under axial compression load conditions and torsional load conditions. **(A)** Change in ALLSE in the LP and RP constructs under different axial compression load values **(B)** Statistical analyses of ALLSE for the LP and RP constructs under all axial compression load values (*p* > 0.05) **(C)** Change in ALLSE in the LP and RP constructs under different torsional load values **(D)** Statistical analyses of ALLSE for the LP and RP constructs under all torsion load values (*p* > 0.05).

**FIGURE 4:**
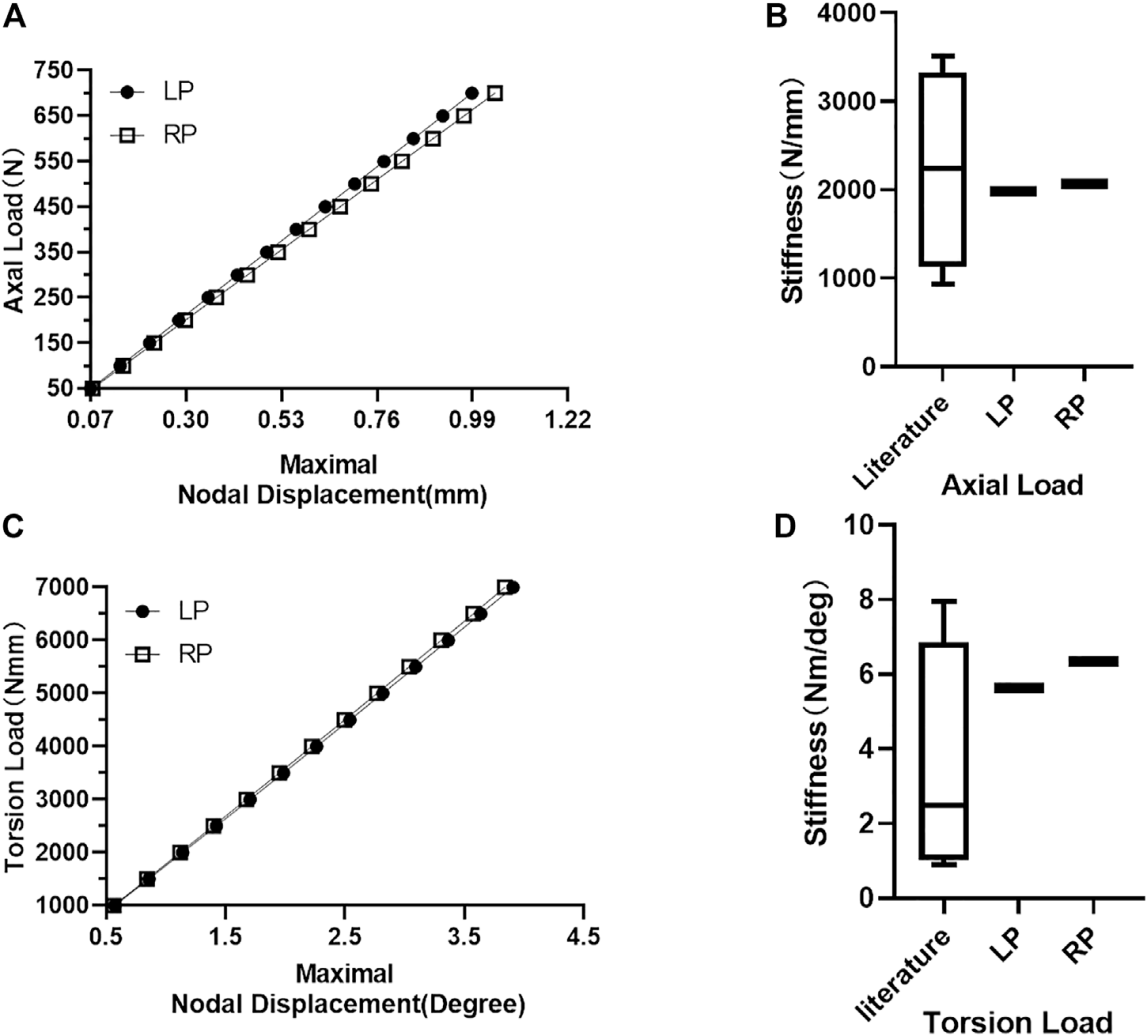
Maximum node displacement and construct stiffness. **(A)** Constructs’ maximum nodal displacement increases with load under axial compression load conditions. **(B)** Comparison of LP and RP construct axial compression stiffness with the stiffness values reported in the literature. **(C)** Constructs’ maximum nodal displacement increases with load under torsional load conditions. **(D)** Comparison of the torsional stiffness of LP and RP construct with the stiffness of previous studies.

In the FE simulation, the strain energy represented the carrying capability of the entire structure. Figure 3 shows the strain energy curves of all constructs as the load increases. No statistical difference is seen in strain energy as the axial compression load (P>0.05) and the torsion load increase (P>0.05) between the LP and the RP constructs. Figures 4A,C shows the maximal value of the nodal displacement of each construct under all loads. There were no statistical differences in maximal nodal displacement (P>0.05) as the axial compression load increased (0.99 and 1.04 mm in RP fixation and LP fixation under the axial loading condition of 700 N, respectively); similar results were obtained for torsion load (P>0.05) with maximal nodal displacements of 3.84° and 3.91° under 7000 N·mm torque, respectively. The stiffness of the RP construct was 4.23% lower than that of the LP construct (1984.22 N·mm compared to 2071.85 N·mm; Figure 4B). The torsional rigidity of the RP construct was 12.6% higher than that of the LP construct (6.34 Nm/deg compared to 5.63 Nm/deg; Figure 4D). For model validation, the stiffness derived from the FE model was highly consistent with the experimental stiffness found in previous studies. (Cristofolini and Viceconti, 2000; Stoffel et al., 2004; Snow et al., 2008; Gardner et al., 2010; Yenna et al., 2011; Liu et al., 2017; MacLeod et al., 2018).

### Construct Safety Assessment

The comparison of the plate designs of the RP and LP constructs is shown in Figure 1C. The volume of the plate in RP construct was 3159.61 ${\text{mm}}^{3}$ and that in the LP construct was 4481.82 ${\text{mm}}^{3}$ . The average stress on the plates generated by the RP construct under maximum axial compression loading conditions was 31.57 MPa, and under maximum torsion, 80.03 MPa. The average stress of the plates generated by the LP construct was 27.63 and 122.49 MPa, respectively, under maximum axial compression and torsional loads.

**FIGURE 5:**
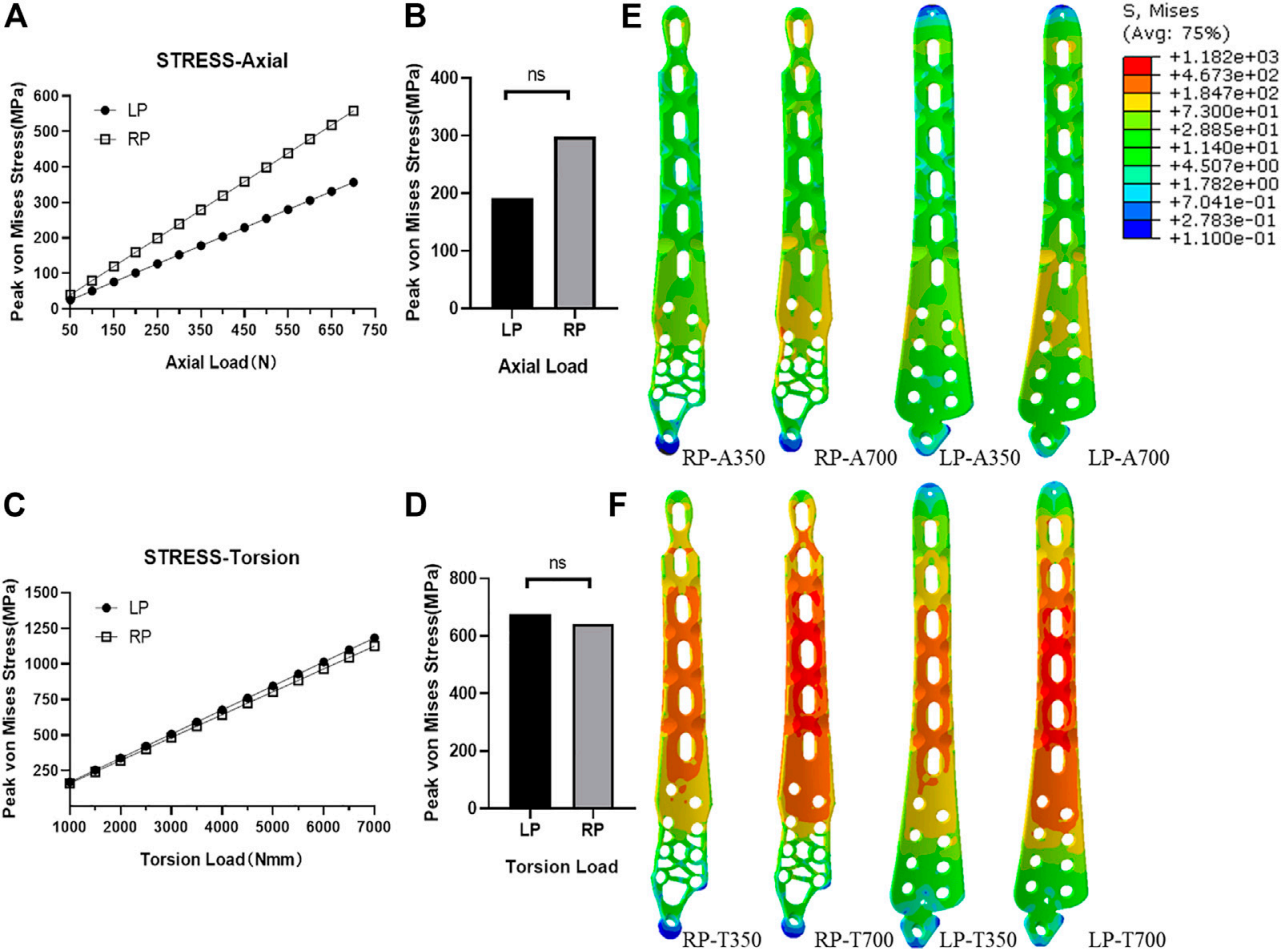
Plate stress analyses. **(A)** Peak stress with difference load values for two kinds of plates under axial compression conditions. **(B)** Statistical analyses of peak stress of the load values of LP and RP plate under axial compression conditions (*p* > 0.05). **(C)** Peak stress with difference load values for two kinds of plates under torsion conditions. **(D)** Statistical analyses of peak stress of the load values of LP and RP plate under torsion conditions (*p* > 0.05). **(E)** Stress distribution of LP and RP plate under axial compression load conditions with a standard physiological load and twice that in the physiological load case. **(F)** Stress distribution of LP and RP plate under torsional conditions with a standard physiological load and twice that in the physiological load case.

The peak values of the von Mises stresses on the plates under all loads are presented in Figure 5. The maximal stresses of the plates in the RP and LP constructs were 558.30 and 356.57 MPa (P>0.05) in the axial compression and 1125.18 and 1182.40 MPa (P>0.05) in the torsional loading conditions, respectively.

Nevertheless, when the axial compression load was less than or equal to the physiological load (350 N), the observed peak stress of the plates in the constructs was much smaller than the yield stress of the Ti6Al4V material. Under the torsional physiological load (3500 N·mm), the peak stress of the plate in the RP construct was 562.6 MPa, and the peak stress of the plate in the LP construct was 591.2 MPa. In the transmission of the axial compression load, when the load increased to 700 N, the element stress of plates was still much lower than three-quarters of the yield stress of the Ti6Al4V material (Figures 5E,F).

**FIGURE 6:**
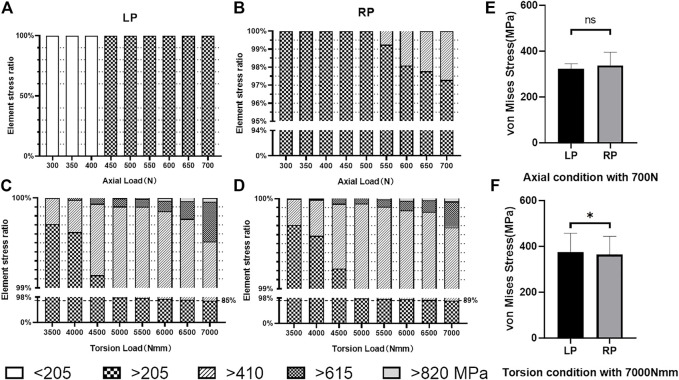
Elemental stress distribution. **(A)** Stress distribution under load values of the LP plate element in axial compression load conditions. **(B)** Stress distribution under load values of the RP plate element in axial compression load conditions. **(C)** Stress distribution under load values of the LP plate element in torsional load conditions. **(D)** Stress distribution under load values of the RP plate element in torsional load conditions. **(E)** Number of the elements which exceeds the allowable stress of 275 MPa under axial compression conditions in twice that of the physiological load case (*p* > 0.05). **(F)** Number of elements which exceeds the allowable stress of 275 MPa under torsion conditions in twice that of the physiological load case (*p* < 0.05).

Furthermore, the allowance stress of the titanium alloy was adopted for safety assessment, following previously published work. (Yuping Deng, 2020). The allowance stress can be calculated by $\;\left[\sigma \right]=\frac{\sigma_{\gamma }}{n}$ , and we selected two allowable stresses according to the material characteristics, strict $\left[\sigma_{1}\right]=275\;MPa$ and secondary $\left[\sigma_{2}\right]=410\;MPa$ , as the max-safe stress and min-safe stress of the plate, respectively—that is, the reference for the allowable stress. Statistical analyses were performed on the part of the plates’ element stress exceeding 275 MPa in the LP and the RP constructs under doubled physiological load groups (Figure 6). For the elements of the plate within the allowed stress range, the average stress of the elements in the LP construct was 322.68 MPa, and in the RP construct, it was 337.48 MPa (P>0.05) . The average stress of the elements of the plate in the LP construct and the RP construct under torsion conditions was 375.12 and 364.21 MPa, respectively (P>0.05) (Figure 6F).

## Discussion

Due to fractures, surgery, and plate implantation, the soft tissue conditions around blood vessels change, which leads to changes in the external mechanical environment and increasing stress on the blood vessels (Liu et al., 2016). On the other hand, changes in the vascular diameter can cause changes to the hemodynamics as well as vascular endothelial damage (Hinderliter et al., 2002; Jou et al., 2005; Liu et al., 2016; Cuomo et al., 2017). Mechanical factors work together inside and outside of the blood vessels, activating the endothelial cells and smooth muscle cells, leading to further changes in blood vessel morphology (Pfisterer et al., 2014; Cuomo et al., 2017). This is consistent with commonly observed adverse events during the clinical treatment of fractured distal tibia, such as poor wound healing, delayed wound healing, and delayed fracture healing (Guo and DiPietro, 2010; Gantwerker and Hom, 2012).

We developed an iterative topological optimization algorithm to design an optimized plate that reduced the overall plate volume by 30% compared to the prototype plate without changing the maximum thickness. According to our analyses of the theoretical model (Figures 2B,C), the method of continuum structure optimization reduced the volume of the subcutaneous space occupied by the internal implant, specifically LP. This method can reduce the strain of the tension direction ε and that in the vertical direction $\;\varepsilon^{\#}$ of the soft tissue, which in theory causes decreased stress acting on the blood vessels in soft tissue. As the effect on the vascular diameter decreases, the effects on blood flow velocity, flow rate, and strain on the vascular elements caused by these changes in hemodynamics also decreases (Liu et al., 2001; Berman et al., 2020). Clinical research reports on the treatment effects of lightweight design plates are in good agreement with the theoretical analyses found in the present study (Carter et al., 1998; Borens et al., 2009; Chen et al., 2021), namely, that the impact of the internal plant on the soft tissues around the surgical area is alleviated. Due to the large differences in load-bearing capacity, mechanical behavior, and stress conduction of the various components of the skin and soft tissues of the distal tibia under physiological conditions, opinions on the distal tibia of the soft tissues and blood fluid-solid coupling simulation have always been based on algorithms and theoretical models that are almost impossible to perfectly validate (Alberto Figueroa et al., 2009; Krittian et al., 2010; Liu et al., 2020; Humphrey, 2021) and are almost irreproducible in the laboratory. On other hand, there are many factors that affect fracture healing, and the fixation effects of the implant on the fracture block are considered the primary relevant factor (Davis et al., 1990; Norris et al., 2018; Hu et al., 2019; Li et al., 2020).

What’s more, we investigated the constructs’ maximum nodal displacement and overall construct stiffness at different loads under axial compression and torsional to find out that the difference in fixation effect between RP structure and LP structure is not statistically significant. We assessed that the RP and LP construct are consistent in their ability to fix the fracture block and limit the displacement of the fracture end. On the other hand, in previous research and in clinical practice, it has been found that the damage severity of the implants was closely related to strain energy and stress (Duan et al., 2011; Guastaldi et al., 2020). This study evaluated the stress and strain energy for RP and LP as an evaluation of the safety of the optimized plate (Walker et al., 2020; Zeng et al., 2020). We found no statistically significant differences between the LP and RP plates in terms of model strain energy and maximum nodal displacement. In summary, we have shown that, after topology optimization and redesign, RP reduces volume by 30%, while the effectiveness and safety remain basically unchanged.

We used an effective method that incorporates strength theory and is suitable for metal materials, allowing strict and secondary allowable stresses to estimate the fatigue failure of the internal fixation under loose and strict allowable stress requirements. In the result, the stress of the plates’ elements is greater than the strictly allowable stress $\left(\left[\sigma_{1}\right]=275\;MPa\right)$ and does not statistically significantly differ in the axial compression conditions between the LP and the RP construct, but the torsion does differ. We believe that this result shows that as the load gradually exceeds twice the physiological loading area, a difference is seen in the safety of implants in torsion conditions between the optimized construct RP and the prototype construct LP (Figures 6E,F). In our analyses of the proportions of different stress elements of the plates in the constructs, we found that the overall stress under physiological load in axial compression stress conditions was lower than the secondary allowable stresses $\left(\left[\sigma_{2}\right]=410\;MPa\right)$ (Figures 6A,B), while under torsional stress conditions, the physiological load may be higher than 410 MPa. When the load increases to twice that of the natural physiological load, under torsion conditions, the element stresses of the LP and RP constructs are 85 and 89% below 410 MPa (Figures 6C,D). Combined with the stress cloud diagram (Figures 5E,F), we believe that the stress distribution of RP is more uniform than that of LP. Through experimental analyses of the stress distribution of the plate elements (Figures 6C,D), we conclude that the number of elements exceeding the secondary allowable stress under the torsion load condition accounts for a larger proportion of the total number of elements. According to the fatigue life S-N curve theory, we have reason to believe that the RP construct is expected to obtain a longer fatigue life due to its smaller number of high-stress elements.

The limitations of this study are as follows: Because the structural and mechanical properties of soft tissues under physiological conditions are still controversial, and the changes in the aforementioned conditions under trauma are not clear, this study’s analyses were only theoretical, based on an idealized model of the soft tissue force. The focus of the next stage of our research will be supplementary mechanical analyses based on the nonlinear material properties of soft tissues, and the theory will be verified through existing methods.

Second, this three-dimensional structure, created using optimization algorithms (which is a better scheme regarding the thickness of the plate structure), is still difficult to manufacture through traditional subtractive manufacturing, and further research on additive manufacturing is required.

In general, these experimental results show that use of finite element simulation analysis combined with topology optimization can improve the capacity of soft tissue protection by reducing the volume of the implant while ensuring the safety of RP by leaving only the necessary structure and generating a more uniform stress distribution. We infer that a smaller plate volume could reduce the excessive mechanical stimulation of soft tissues caused by the implants, and the occurrence rate of adverse prognostic events caused by poor mechanical stimulation may decrease.

## Data Availability

The original contributions presented in the study are included in the article/Supplementary Material, further inquiries can be directed to the corresponding authors.
